# Strain Rate Sensitivity of Low Carbon Threaded Steel Rods of Grade 4.6

**DOI:** 10.3390/ma17246228

**Published:** 2024-12-20

**Authors:** Jovan Trajkovski, Robert Kunc

**Affiliations:** Chair of Modelling in Engineering Sciences and Medicine, Faculty of Mechanical Engineering, University of Ljubljana, Aškerčeva c. 6, 1000 Ljubljana, Slovenia; robert.kunc@fs.uni-lj.si

**Keywords:** material characterization, strength parameters, thread failure, numerical simulation, road barriers

## Abstract

Bolt connections are widely used in construction and engineering to securely join structural elements. These connections are essential for distributing loads across components and ensuring that structures can withstand external forces. The planned failure of these bolts is of great importance in steel safety barriers (SSBs), as it can directly influence the height of the guardrail and the working width of the SSB during the vehicle impact, which consequently affects the crash consequences. Therefore, it is of great importance to determine the bolt response until fractures under different strain rates. For that purpose, experimental tensile tests of low-strength steel rods of grade 4.6 were conducted at various strain rates (0.0025–25 s^−1^) until fracture. Test specimens were photographed during the testing, and by means of image processing, input data for calculation of true stresses and strains up to the point of fracture were extracted. Based on the experimental data, material parameters were determined for the Cowper–Symonds model, enabling precise numerical simulations of these connections at various strain rates. A validation study was also performed successfully.

## 1. Introduction

Most of the bolt connections, particularly in structural and mechanical applications, are designed to respond within their elastic loading regime. However, around 10–20% of bolted connections are designed to operate in their plastic loading regime, in which they can potentially experience yielding, permanent deformation, and even failure [1]. During events like earthquakes, vehicle crashes, blast loading, or ballistic impact, products usually experience a single load of high intensity [1]. These sudden, high-magnitude loads can result in severe deformations and stress within a fraction of a second. The combination of load intensity, material properties, and structural geometry may lead to critical failures, human injuries, or even death. Thus, it is crucial to evaluate and enhance the product’s ability to withstand such extreme, short-duration loads or to be able to precisely predict their failure. Experimental examination of such a large number of large-scale physical tests is often expensive and requires interdisciplinary expertise, legal clearances, and additional safety measures. As a result, numerical analysis, especially the Finite Element (FE) analysis, has become a key engineering tool for examination of the structural response. On the other side, an accurate FE analysis requires precise material data that can describe material behavior, not only the elastic but also the plastic response, as well as reliable predictions of fracture initiation, propagation, and final extent—each posing significant challenges in the field.

The response of threaded steel fasteners at higher strain rates has been extensively studied experimentally [1,2,3,4] as well as numerically [1,5,6]. Although constant progress has been made during the last decades, the precise numerical modeling of threaded fasteners is still challenging due to the complexity that involves material nonlinearities (including both strength and fracture models), high-intensity multiaxial loads and usage of multiple groups of bolts. An ideal constitutive equation includes a relatively small number of material parameters, the determination of which requires a minimum number of tests, enabling at the same time precise modeling of processes within a wide range of strains, strain rates, and temperatures [7]. The simplest description of the strain rate influence on flow stress is presented by the Cowper–Symonds model [8], which has been widely used to describe the strain rate sensitivity of various materials. Mahalle et al., 2019 [9] determined for Inconel 718 alloy, and Noh et al., 2016 [10] determined the parameters for Al 1100-O by applying the inverse method and a genetic algorithm. In studies by Škrlec, Kocjan et al., and 2024 Škrlec, Panić et al., 2024 [11,12] different test methods and material parameters determination techniques were used to study the behavior of the SZBS800 steel. Yang et al., 2024 [13] examined the rate-dependent behavior of high-strength steel M12 bolts of strength grades 8.8 and 12.9, delivering the material parameters of the Cowper–Symonds model, while Warren et al., 2022 [1] presented a great overview of studies related to experimental and numerical modeling of the dynamic fracture of bolts. A more complicated and uncoupled model that has also been widely used and additionally includes a thermal softening component is the Johnson–Cook strength [14] and fracture model [15].

Road safety barriers play a crucial role in ensuring traffic safety by keeping vehicles on their lane during a collision. In the case of steel safety barriers (SSBs), a collision leads to significant deformation, often accompanied by the failure of bolts connecting the guardrail to the posts. The planned failure of these bolts is of great importance, as it can influence the height of the guardrail and the working width of the barrier, which in turn affects the load parameters experienced by the passengers. Therefore, designing these bolt connections to respond in the plastic range is essential for creating safe SSB systems [6,16].

Bolts, fasteners, or threaded rods of the 4.6 strength class are low to medium-strength fasteners commonly used in non-critical general-purpose applications where high tensile strength is not required. These bolts are typically made from mild steel, providing good ductility but lower strength compared to higher-grade bolts such as 8.8 or 10.9. This paper presents the experimental results of tensile tests of M6 threaded rods of strength class 4.6 performed under different strain rates. The test samples were photographed during the testing, and the photographs were analyzed through edge detection between a specimen and its contrasting background, based on which data on the change in length as well as diameter of the specimen were obtained. With these data and appropriate values of the measured force, the true stress–strain curves were constructed until fracture. This paper describes in detail the process of determining the true stress strain curves until fracture and delivers the Cowper–Symonds material parameters that describe the strain rate influence on flow stress for threaded rods of class 4.6. The results presented in this paper will enable a more precise numerical response of strength class 4.6 bolts in the modeling of more complex, highly nonlinear numerical processes which can potentially involve nonlinearities of all kinds (material, geometric, contact, and boundary) such as vehicle impacts on steel safety barriers or any other structures where bolts of this strength class are used.

## 2. Experiments and Methods

### 2.1. Tests

**Figure 1:**
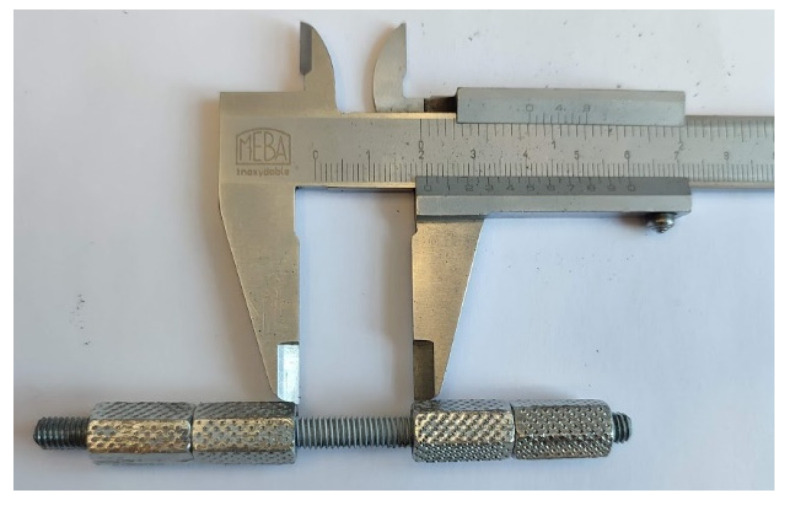
Specimen geometry.

**Figure 2:**
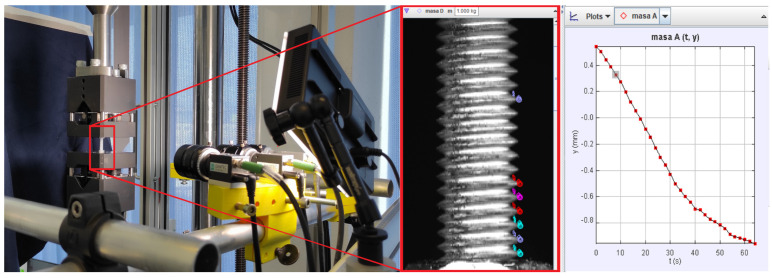
Taking a photograph of a specimen and image processing.

Uniaxial tensile tests at room temperature were performed on a multiaxial dynamic test rig StepLAB UD 020 (STEP Engineering S.r.l., Treviso, Italy) according to ISO 6892-1 [17]. The total length of all samples was 90 mm ± 0.1 mm, while the length between the nuts was 20 mm ± 0.1 mm (Figure 1). A total of 18 samples were tested under six different strain rates (Table 1). For the purpose of constructing the true stress–strain curve after the onset of necking, the specimens were photographed during the testing. In order to increase the contrast between the background and the specimen, the black textile was used while the specimen was lit using a 500 W reflector (Figure 2).

### 2.2. Material

The ISO 898 standard [18] defines physical and mechanical properties for fasteners that range from M1.6 to 100 in diameter. The chemical composition of 4.6 strength class bolts, screws, and threaded rods, as well as the basic mechanical properties, are defined in Table 2 and Table 3 accordingly.

### 2.3. Video Analysis

During this study, a 3D Digital Image Correlation (DIC) system with three cameras by Dantec Dynamic was tested to capture the deformation process of the threaded rods. However, due to the complexity of the thread rod geometry, we were not able to use the advanced capabilities of the DIC software. Throughout this phase, the DIC cameras were primarily used to capture the deformation process of the specimen using the advanced optical capabilities of the DIC cameras, which provided real-time data on changes in the length and sample diameter. We used a 50 mm focal length for the cameras, and the videos were captured between 0.1 and 250 fps (Table 1). Every video has a resolution of 1588 × 720. This configuration enabled simultaneous monitoring of the sample deformation process.

**Figure 3:**
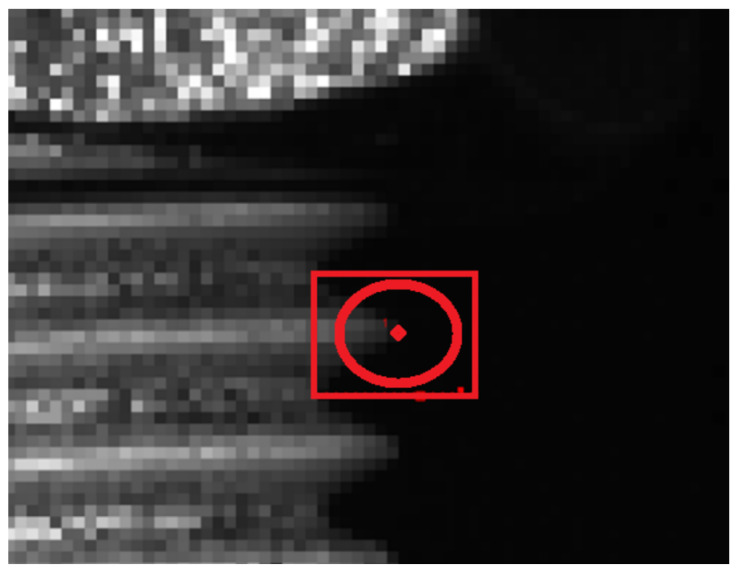
Definition of the tracking pattern.

For the analysis of the recorded videos, we used the 6.2.0 version of the Tracker software, which is built on the Open-Source Physics (OPSs) Java framework (Tracker, 2024). The program allows for automatic tracking of a pattern within a predefined area, which is ideal for tracking thread peaks (Figure 3). Before tracking analysis was run, the initial and final frames were defined along with the time step. The initial frame was defined as the one just before a visible movement was observed, and the final frame was just before the specimen fracture. We defined the pattern to track (thread pick in Figure 3) and the search area for which we searched for the best match in subsequent images.

Before tracking, we also need to set three additional parameters: Evolution rate, Tether, and Automark. The evolution rate defines how quickly the pattern will adjust to color and shape changes over time. An evolution rate of 0% means no evolution occurs (the reference image remains constant), while a 100% rate means the pattern image is completely replaced after each step. High evolution rates allow for changes to be tracked faster. However, precision is also lost faster [19,20]. The Tether adjusts how much the pattern should evolve relative to the key image. This parameter allows us to set the extent of acceptable image change. It is useful when a high evolution rate is set. Higher percentages restrict allowable changes, which helps with faster tests. It works by comparing the initial image with the image before the current step [19]. Automark sets the minimum match rating required for the program to automatically mark a point. The lower the Automark, the sooner auto-marking is achieved. However, this increases the chance of errors. In our case, we set this parameter to the highest value, which is 10/10 [19,20]. After testing and setting all parameters, automatic tracking was initiated. If the match rating is adequate, the point will be tracked automatically until the final frame. In some cases, when the deformation of the sample progresses, the automatic tracker cannot reach the match rating, and the program will indicate an error. We can then manually reduce the matching criteria by reducing the Automark to a smaller value or repeat the search by shifting the search area. Each point can always be manually adjusted, giving us complete control over the marking process.

**Figure 4:**
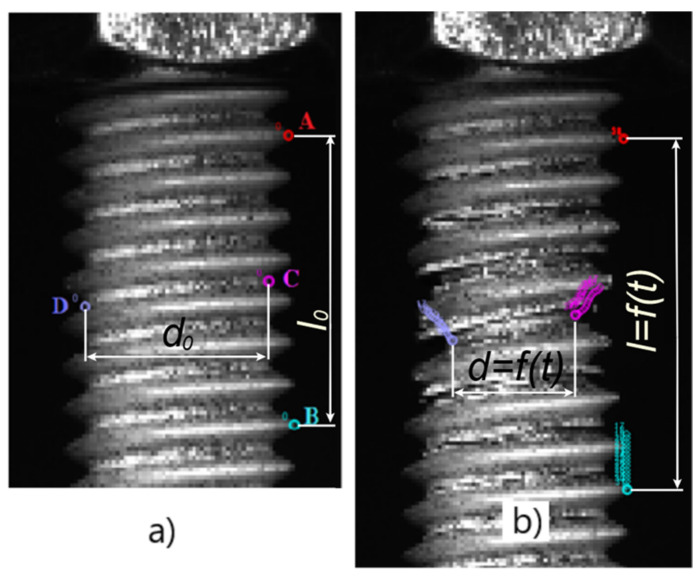
Video analysis of the threaded rods: (**a**) undeformed state, (**b**) state after fracture.

In most of our analyses, we set the evolution rate to 20% and increased it only for tests with the highest deformation rate, up to a maximum of 25%. The Tether was set to 10% for all tests. The Automark value was initially set to its maximum value of 10 for all tests and reduced to 9 or 8 in the cases when automatic tracking stopped. For each test, we followed four points: A, B, C, and D (Figure 4). We identified two points (A and B in Figure 4) on the thread peaks located two to three thread pitches from the fracture location. By tracking these two points, we were able to calculate the elongation. Points C and D were used to determine the reduction in the bolt diameter. An image just before the fracture with the measured points is shown in Figure 4. After video analysis and data collection, we proceeded with the evaluation of the strain rate influence on maximum stress as well as with the calculation of true stress strain curves.

### 2.4. Material Characterization

**Figure 5:**
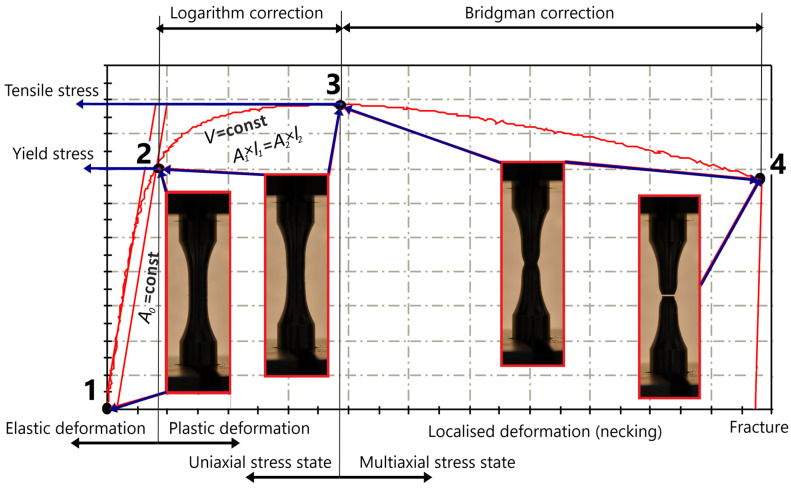
Stress–strain regions of tensile specimens.

Most machine parts and structures are designed to operate and respond to external loads within the elastic deformation range. Under these loads, the resulting deformations are completely elastic, which means the loaded parts return to their original undeformed state once the load is removed. In a uniaxial standard tensile test, this state corresponds to the region 1–2 in Figure 5. In such cases, the elastic modulus *E* and Poisson’s ratio *ν*, combined with an appropriate safety factor, provide sufficient material data for designing/dimensioning or numerical analysis of structures. However, designing structures in the elastic range can lead to very massive constructions (e.g., vehicle chassis in crash scenarios), which forces us to design for maximum efficiency under such conditions. Additionally, most manufacturing processes (forging, rolling, shaping) occur in the plastic region of the material response, making it essential to accurately predict material response beyond the yield point (point 2 in Figure 5).

As is well known, the engineering stress–strain curve is based on the initial geometry (initial area *A_0_* and initial length *L*_0_) of the specimen (Equation (1)) and is therefore only valid in the elastic range of the material response (1–2, Figure 5). The actual stress–strain curve can be determined up to the beginning of necking or the maximum force (2–3) by correcting the engineering curve using logarithmic equations (Equation (2)).

(1) $$\sigma_{eng}=\frac{F}{A_{0}}\;\;\;\;\;\;\;\varepsilon_{eng}=\frac{∆L}{L_{0}}$$

(2) $$\sigma_{T}=\sigma_{eng}\left(\varepsilon_{eng}+1\right)\;\;\;\;\;\;\varepsilon_{T}=ln\frac{L}{L_{0}}$$

After the maximum measured force (point 3 in Figure 5), the process of the so-called “necking” begins [21]. The deformation becomes localized, and therefore, a multiaxial stress state occurs in the material (Equation (2) are no longer valid after point 3), necessitating correction of the measured force (Equation (3)). An even more challenging engineering step is the accurate prediction of possible damage initiation, its progression, and final size under external loads.

(3) $$\sigma_{T}=\frac{F}{\left(\frac{d^{2}\pi }{4}\right)\left(1+\frac{4R}{d}\right)ln\left(1+\frac{d}{4R}\right)}\;\;\;\;\;\;\;\varepsilon_{T}=2ln\frac{D_{0}}{D}$$

The mechanical properties of materials largely depend on changes in temperature, strain rate, loading mode, and stress state complexity. Therefore, an appropriate constitutive material model is needed to effectively describe the actual state of the material. The simplest material model to describe material response under large deformations and varying strain rates is the bilinear model, which considers the effect of strain rate on flow stress based on the Cowper–Symonds model [8] for which material parameters were determined in this study. More advanced material models can also be used for more accurate predictions.

(4) $$\sigma_{d}=\sigma_{s}\left[1+{\left(\frac{\dot{\varepsilon }}{C}\right)}^{1/p}\right]$$

In Equation (4), σ*_S_* represents the static and σ*_d_* dynamic stress, *C* and *p* are material parameters determined from the experiments, and *ἑ* represents the strain rate.

### 2.5. Material Parameters Identification

Various strategies for identifying material parameters are extensively discussed in the literature. These strategies are generally categorized as either coupled or uncoupled [22,23], depending on how specific parameters are determined. An alternative classification is based on the calibration approach used, ranging from direct identification methods to more sophisticated optimization techniques [24], such as the least-squares method, weighted multi-objective optimization [25,26], and genetic algorithms [27]. Inverse methods are also commonly applied to determine material parameters or to derive the true stress–strain curve [10,25]. The literature also presents a variety of experimental setups, sample geometries, and data processing techniques, all of which can result in different material parameters for the same material. Hence, careful attention is needed when performing material parameter identification to ensure accuracy and consistency.

### 2.6. Model Validation

**Figure 6:**
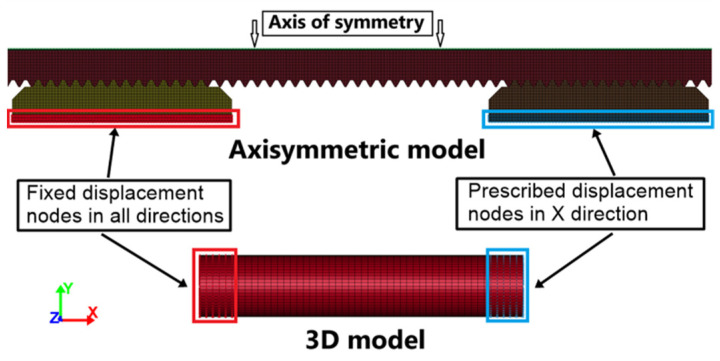
Description of the axisymmetric and 3D numerical models.

After determining the parameters of the Cowper–Symonds model, the experimental tests were numerically simulated by means of the LS-DYNA finite element explicit code, using preliminarily determined material parameters of the Cowper–Symonds model. For that purpose, two numerical models were prepared: a simplified 3D model and a more detailed 2D axisymmetric model (Figure 6). Full 3D numerical simulations were performed representing the samples with fully integrated eight-node solid elements in the 3D model, while axisymmetric volume-weighted solid elements were used in the 2D-axisymmetric model. Around fifty elements across the diameter represent the sample mesh, leading to an element size of 0.12 mm in the simplified 3D model, while a 0.05 mm mesh size was used in the 2D axisymmetric model. In order to decrease the calculation time, simulations were performed using the mass scaling technique, carefully monitoring the ratio of the kinetic and internal energy. A mesh sensitivity study was also performed successfully.

## 3. Results and Discussion

### 3.1. True Stress–Strain Curves

**Figure 7:**
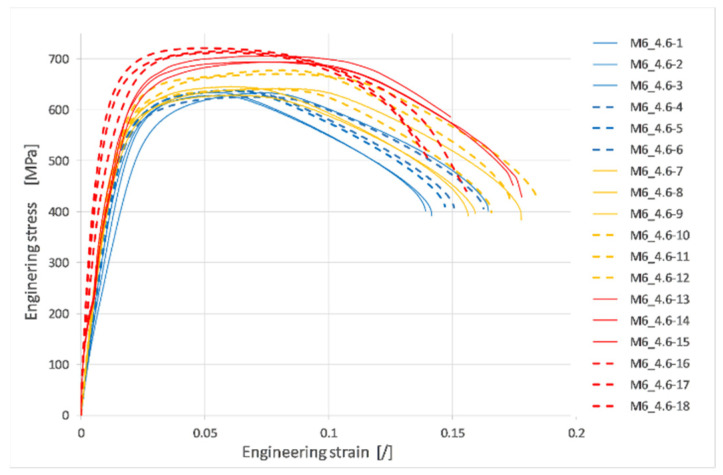
Engineering stress–strain curves of M6 threaded rods of the 4.6 strength class under different strain rates.

**Figure 8:**
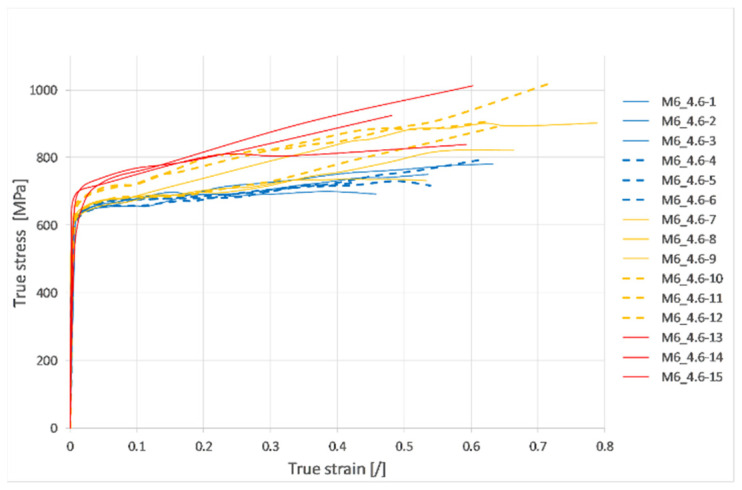
True stress–strain curves of M6 threaded rods of the 4.6 strength class under different strain rates.

The engineering stress strain curves based on the crosshead displacement for all tests are shown in Figure 7. However, those curves represent rough data that also include the stiffness of the test rig and, therefore, cannot be used as such. Combining the force–time curve from the load cell, length, and diameter changes from the video analysis and using Equations (2) and (3), the true stress strain curves shown in Figure 8 were constructed until fracture.

**Figure 9:**
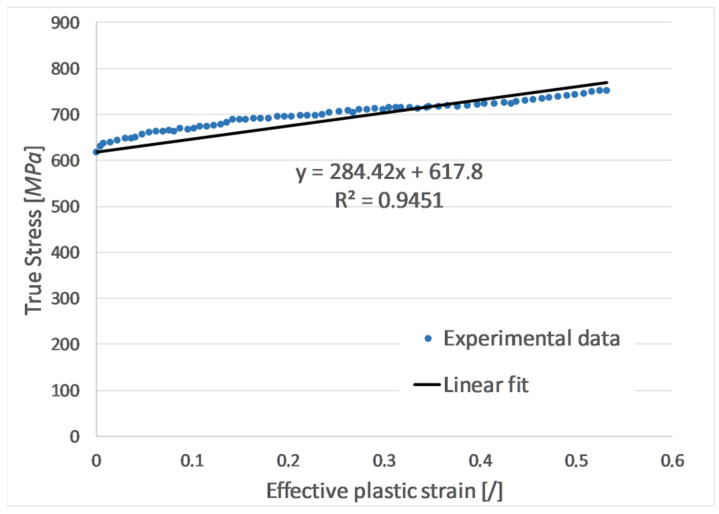
Tangent modulus determination.

From the average three true stress–strain curves of the reference group, the effective plastic strain was obtained simply by extracting the elastic strain of 0.2% starting at the point of Yield stress (σ_y_ = 617.8 MPa). To present the obtained results more effectively for direct use by finite element analysts, the curve of effective stress versus effective plastic strain is provided in tabular form (Table 4). This format is commonly used as input data for most material models that are used in explicit finite element codes (Example: *MAT_PIECEWISE_LINEAR_PLASTICITY). Figure 9 shows the determination of the tangent modulus of the material (*E_t_* = 284.42 MPa) by a linear approximation of the true stress-effective plastic strain curve, with the intercept on the *y*-axis set at the yield stress.

Although it represents only a simplification of the multilinear curve defined in Table 4, the coefficient of determination (*R*^2^ = 0.9451) demonstrates a strong correlation between the linear fit and the experimental data.

### 3.2. Cowper–Symonds Materials Parameters Identification

**Figure 10:**
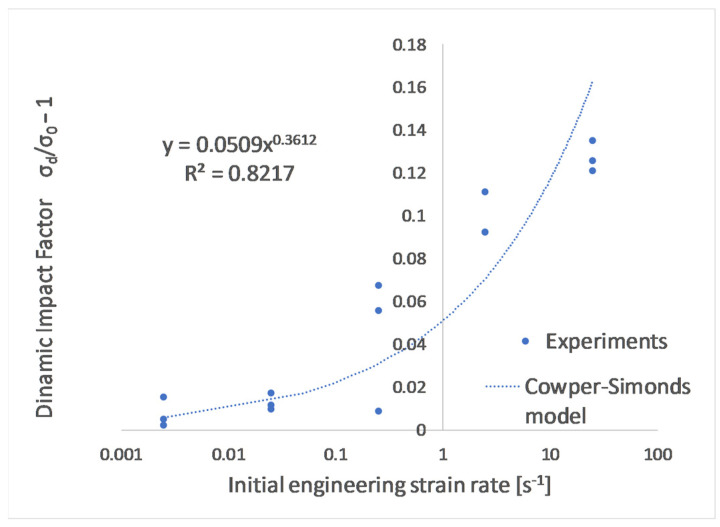
Identification of Cowper–Symonds material parameters.

The experimental ratios between the dynamic tensile stress from tests conducted at higher strain rates (0.025 s^−1^ to 25 s^−1^) and the average tensile stress from three reference tests performed at the lowest strain rate of 0.0025 s^−1^ are presented in Figure 10, marked with a blue dot. For the material identification procedure, an uncoupled method was adopted, involving the direct fitting of the Cowper–Symonds model (Equation (4)) to the experimental results using the least squares method. In this analysis, the Cowper–Symonds model coefficients were determined as *C* = 3806.41 s^−1^ and *p* = 2.7685. The coefficient of determination was higher than R^2^ = 0.8217, indicating a strong fit.

The values of the material parameters are highly dependent on the strain-rate range of testing, type of experiments performed, sample geometries, and data processing techniques applied for their determination. This will undoubtedly lead to different material parameters for the same material [7], which is often proven in the literature. According to the comparison study for some high-strength steels performed by [12], the values for the *C* parameter span many orders of magnitude, ranging from 2.68 [28] to 86,700,000 [29]. The range for the *p* parameter was found to be between 1.31 and 30.4 (with most of the values below 6.7). In our case, the material parameters for the Cowper Symonds model for the particular material of strength class 4.6 do not exist in the literature, and therefore, direct comparison is not currently possible. However, the commonly used parameters *C* and *p* (often denoted as *D* and *q*) for mild steel are 40.4 s^−1^ and 5, respectively, which provide satisfactory similarity.

## 4. Validation Study

**Figure 11:**
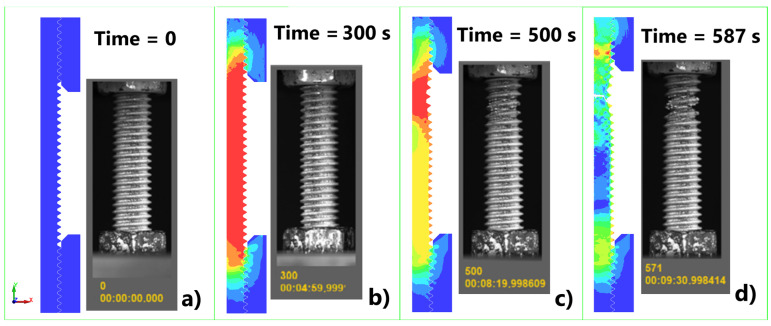
Visual comparison between the experimental and numerical tests: (**a**) t = 0 s, (**b**) t = 300 s, (**c**) t = 500 s, (**d**) time at fracture.

**Figure 12:**
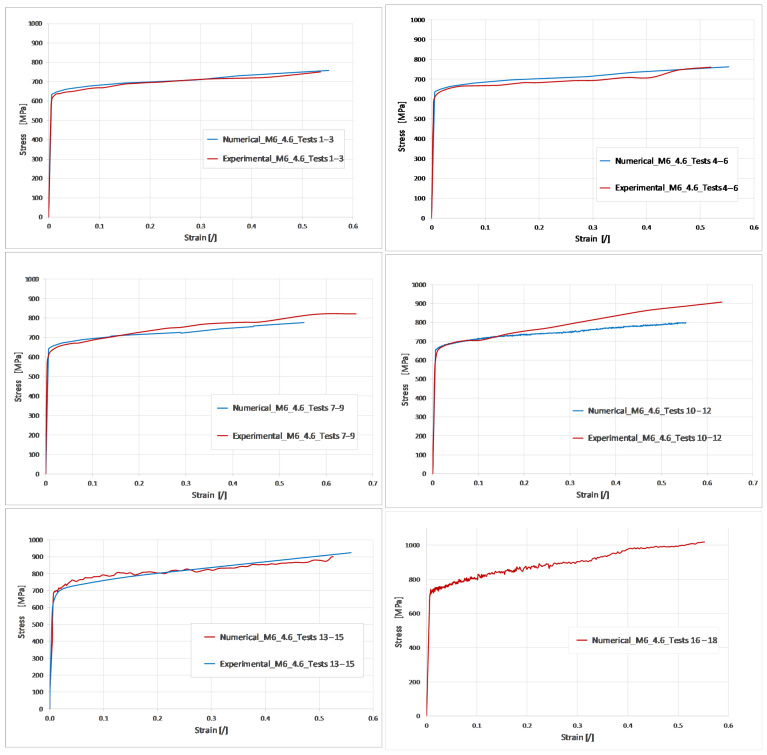
Comparison of experimental and numerical results.

After the determination of the Cowper–Symonds material parameters, a simulation of the tensile test was performed in LS-DYNA. Figure 11 presents a visual comparison between the experimental and numerical tests 1–3 (Table 1) in different comparison times. It can be noticed that the necking process of the sample is well captured (Figure 11b), while the time at fracture slightly differs. The stress–strain curves from the simulation were compared with the experiment (Figure 12), showing good agreement through the field of deformations. The results comparison for tests 16–18 is missing due to limitations of the equipment used and the insufficient number of images obtained for data processing.

**Figure 13:**
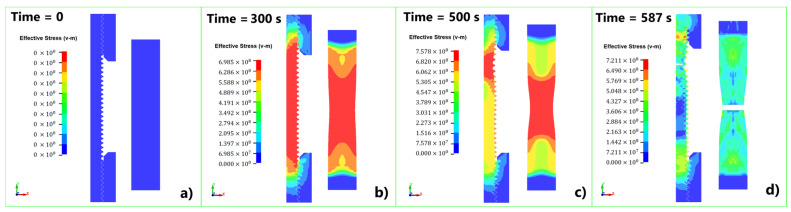
Results comparison between the axisymmetric and 3D model: (**a**) t = 0 s, (**b**) t = 300 s, (**c**) t = 500 s, (**d**) time at fracture.

Figure 13 shows the comparison between the simplified 3D model and 2D axisymmetric model at different comparison times. It can be noticed in this Figure that the 3D model captures the deformation process very well, although it has simplified geometry and a larger element size compared to the 2D axisymmetric model.

## 5. Conclusions

In this study, tensile tests on low-strength M6 threaded rods of the 4.6 strength class were conducted across a range of strain rates from 0.0025 s^−1^ to 25 s^−1^ to investigate their performance under dynamic loading conditions relevant to steel safety barriers (SSBs). The analysis highlighted the importance of planned bolt failure within SSB systems, demonstrating its influence on critical strength model parameters, which are crucial for vehicle impact safety. Through image-based measurement of specimen deformation, true stress–strain data were generated, leading to precise material characterization. Cowper–Symonds model parameters were derived, enabling realistic simulations of the threaded rod behavior under different strain rates. This approach provides a valuable foundation for enhancing SSB safety through reliable numerical simulations, thus reducing the need for physical tests. The validation study confirmed the robustness of the model, suggesting its potential for broader application in structural safety assessments where bolt response of class 4.6 under extreme loading is critical. However, material parameters were obtained using limited experimental data under relatively low strain rates, which means the dynamic response of threaded rods or bolts should be further investigated to cover the response of these bolts in more severe conditions under higher strain rates characteristic for blast loading of structures.

## Figures and Tables

**Table 1:** Tests.

Sample No.	File Name	Head Speed	Engineering Strain Rate	Acquisition Sampling	DIC Sampling
	4.6_xx	[mm/s]	[s^−1^]	[Hz]	[Hz]
1	4.6_01	0.005	0.00025	5	0.1
2	4.6_02	0.005	0.00025	5	0.1
3	4.6_03	0.005	0.00025	5	0.1
4	4.6_04	0.05	0.0025	50	1
5	4.6_05	0.05	0.0025	50	1
6	4.6_06	0.05	0.0025	50	1
7	4.6_07	0.5	0.025	500	10
8	4.6_08	0.5	0.025	500	10
9	4.6_09	0.5	0.025	500	10
10	4.6_10	5	0.25	5000	100
11	4.6_11	5	0.25	5000	100
12	4.6_12	5	0.25	5000	100
13	4.6_13	50	2.5	50,000	250
14	4.6_14	50	2.5	50,000	250
15	4.6_15	50	2.5	50,000	250
16	4.6_16	500	25	200,000	/
17	4.6_17	500	25	200,000	/
18	4.6_18	500	25	200,000	/

**Table 2:** Tests Chemical composition of carbon steel thread rods of grade 4.6.

Strength Class or Grade	Material and Heat Treatment	Chemical Composition (Analysis of the Product) %					Min. Hardening Temperature °C
		C		P	S	B	
		Min	Max	Max	Max	Max	
4.6	Carbon steel	-	0.55	0.050	0.060	Not specified	-

**Table 3:** Tests Basic mechanical properties of carbon steel thread rods of grade 4.6.

Class/Grade	Material	Proof Stress	Yield Strength (Min.)	Tensile Strength (Min.)	Core Hardness (Rockwell)
4.6	Low- or medium-carbon steel	225 MPa	240 MPa	400 MPa	B67-95

**Table 4:** Effective plastic strain vs. effective stress curve data.

Effective Plastic Strain [/]	Effective Stress [MPa]
0.0000	617.8
0.0033	631.6
0.0112	643.5
0.0310	658.7
0.0760	674.5
0.1445	689.8
0.2872	705.6
0.3725	727.5
0.5263	751.2

## Data Availability

The original contributions presented in this study are included in the article. Further inquiries can be directed to the corresponding author.

## References

[1] Warren M., Antoniou A., Stewart L. (2022). A review of experimentation and computational modeling of dynamic bolt fracture. J. Constr. Steel Res..

[2] Cao Z., Zhang F., Zhang D., Yu Y., Li L., Guo X. (2021). Failure mechanisms of bolted flanges in aero-engine casings subjected to impact loading. Chin. J. Aeronaut..

[3] Fransplass H., Langseth M., Hopperstad O.S. (2011). Tensile behaviour of threaded steel fasteners at elevated rates of strain. Int. J. Mech. Sci..

[4] Mouritz A.P. (1994). Failure mechanisms of mild steel bolts under different tensile loading rates. Int. J. Impact Eng..

[5] Fransplass H., Langseth M., Hopperstad O.S. (2013). Numerical study of the tensile behaviour of threaded steel fasteners at elevated rates of strain. Int. J. Impact Eng..

[6] Schauwecker F., Moncayo D., Middendorf P. (2022). Characterization of high-strength bolts and the numerical representation method for an efficient crash analysis. Eng. Fail. Anal..

[7] Trajkovski J., Kunc R., Pepel V., Prebil I. (2015). Flow and fracture behavior of high-strength armor steel PROTAC 500. Mater. Des..

[8] Cowper G., Symonds P. (1957). Strain Hardening and Strain-Rate Effects in the Impact Loading of Cantilever Beam.

[9] Mahalle G., Kotkunde N., Gupta A.K., Singh S.K. (2019). Cowper-symonds strain hardening model for flow behaviour of inconel 718 alloy. Mater. Today Proc..

[10] Noh H.G., Lee K., Kang B.S., Kim J. (2016). Inverse parameter estimation of the Cowper-Symonds material model for electromagnetic free bulge forming. Int. J. Precis. Eng. Manuf..

[11] Škrlec A., Kocjan T., Nagode M., Klemenc J. (2024). Modelling a Response of Complex-Phase Steel at High Strain Rates. Materials.

[12] Škrlec A., Panić B., Nagode M., Klemenc J. (2024). Estimating the Cowper–Symonds Parameters for High-Strength Steel Using DIC Combined with Integral Measures of Deviation. Metals.

[13] Yang S., Zhu Y., Zhang R., Zhao Y., Yang H. (2024). Rate-dependent behaviour of high-strength steel bolts. J. Constr. Steel Res..

[14] Johnson G.R. Materials characterization for computations involving severe dynamic loading. Proceedings of the Army Symposium on Solid Mechanics.

[15] Johnson G.R., Cook W.H. (1985). Fracture characteristics of three metals subjected to various strains, strain rates, temperatures and pressures. Eng. Fract. Mech..

[16] Borovinšek M., Vesenjak M., Ren Z. (2013). Improving the crashworthiness of reinforced wooden road safety barrier using simulations of pre-stressed bolt connections with failure. Eng. Fail. Anal..

[17] (2009). Metallic Materials—Tensile Testing—Part 1: Method of Test at Room Temperature.

[18] (2013). Mechanical and Physical Properties for Metric Fasteners Made of Carbon Steel and Alloy Steel—Part 1: Bolts, Screws and Studs with Specified Property Classes—Coarse Thread and Fine Pitch Thread.

[19] Brown D., Christian W. Simulating what you see: Combining computer modeling with video Modeling. Proceedings of the Mptl 16—Hsci 2011.

[20] (2024). Tracker. https://physlets.org/tracker/.

[21] Bridgman P.W. (1964). Studies in Large Plastic Flow and Fracture.

[22] Majzoobi G.H., Freshteh-Saniee F., Faraj Zadeh Khosroshahi S., Beik Mohammadloo H. (2010). Determination of materials parameters under dynamic loading. Part I: Experiments and simulations. Comput. Mater. Sci..

[23] Majzoobi G.H., Khosroshahi S.F.Z., Mohammadloo H.B. (2010). Determination of materials parameters under dynamic loading: Part II: Optimization. Comput. Mater. Sci..

[24] Borvik T., Hopperstad O.S., Berstad T., Langseth M. (2001). A computational model of viscoplasticity and ductile damage for impact and penetration. Eur. J. Mech. A/Solids.

[25] Hernandez C., Maranon A., Ashcroft I.A., Casas-Rodriguez J.P. (2011). An inverse problem for the characterization of dynamic material model parameters from a single SHPB test. Procedia Eng..

[26] Milani A.S., Dabboussi W., Nemes J.A., Abeyaratne R.C. (2009). An improved multi-objective identification of Johnson-Cook material parameters. Int. J. Impact Eng..

[27] Škrlec A., Klemenc J. (2017). Parameter identification for a Cowper-Symonds material model using a genetic algorithm combined with a response surface. Eng. Comput..

[28] Stronge W.J., Yu T.X. (1989). Dynamic plastic deformation in strain hardening and strain-softening cantilevers. Int. J. Solids Struct..

[29] Jones N. (1967). Influence of strain-hardening and strain-rate sensitivity on the permanent deformation of impulsively loaded rigid-plastic beams. Int. J. Mech. Sci..

